# New Operation for Cleft Palate; Means of Counteracting the Effects of Chloroform

**Published:** 1856-01

**Authors:** 


### NEW OPERATION FOR CLEFT PALATE.

At the session of the French Academy, on the 26th February, 1855, M. Jules Cloquet read a memoir on a new procedure in cases of "abnormal divisions of certain organs, and particularly in those of the *velum palati.*" M. Cloquet proposes to render available the well-known contractile property of cicatricial tissues, as shown in the various deformities arising from burns and ulcers, by applying to the divided texture a series of cauterizations, commencing at one end of the division, and continuing them throughout its course as rapidly as the wounds, there made, heal and contract, thus obliterating the deficiency. M. Cloquet recounts the difficulties and frequent failure of the operation by suture, and furnishes the details of six cases of *fissured palate* successfully treated by the new method. The objection to the operation is the slowness of the process—but, on the other hand, there are claimed for it certainty, simplicity, and readiness of application to the very youngest subjects, while the process of cure is accompanied with no restraint upon the patient, and does not interfere with her ordinary occupation. A cauterizing agent, the platinum-uin, heated reddish-white by an electric-current, is preferred to the hot iron, as being less likely to alarm the young patient, while it has the advantage of any chemical agent, in being more controllable as to the extent of its action. M. Cloquet proposes, in a second paper, to treat of the application of his method to the cure of ruptures of the peritoneum and of the rectovaginal septum, and of certain varieties of fistula.—*Gazette Medicale.*

### MEANS OF COUNTERACTING THE EFFECTS OF CHLOROFORM.

In the *Gazette des Hopitaux* of September, we find a notice of a session of the Society of Practical Medicine, containing a report by M. Ferrier upon a communication of M. Ludger Lallemant, on the relative value of different agents to neutralize the deadly effects of chloroform.

After detailing the usual phenomena of anæsthesia, as exhibited in man and the lower animals, the report continues:

"Among the means of opposing the poisonous effects of chloroform, experimentists have tried inflation of the lungs with pure oxygen and also with atmospheric air, electricity, irritation of the phrenic nerves, and stimulating the pharynx with caustic ammonia.

"The success obtained by inflating with oxygen has been equalled by the happy results obtained by the use of atmospheric air alone; and authors who have tried with success azote, are convinced that the result should be attributed rather to the irritating action of the gas brought into contact with the walls of the bronchial tubes than to any specific effect in the air cells. They have therefore given the preference to inflation with atmospheric air as the most simple, and of sufficiently easy application by means of a gum-elastic tube provided with a mouth-piece; which, in experiments with dogs, has been introduced into the larynx, or simply into the back part of the mouth in rabbits. The inflation should always be made to alternate with pressure regularly applied to the chest.

"Electricity proposed by MM. Jobert and Abeille has not succeeded in the hands of experimenters. Irritation of the phrenic nerves, suggested by M. Duchenne, of Boulogne, having for its object to restore the regular action of the intercostal muscles, has appeared, on the other hand, to be equally successful with the inflations. The use of caustic ammonia, according to the process of M. J. Galrin, has failed.

"Inflation, as the means of bringing to life the subjects of an excessive employment of anæsthetics, has been oftno benefit except in the cases where it has been used immediately after the cessation of respiration, rarely after the heart has ceased to beat. It is necessary also that inflation should be continued with perseverance and energy, until the normal and spontaneous movements of respiration are fully established.

"It has been remarked, also, that under the influence of anæsthesia the nervous centers and spinal marrow, having become insensible to the touch, are also insensible to the stimulus of the galvanic pile; but the agitation produced by galvanism speedily exhausts the remaining nervous irritability, which very seldom is sufficient to react upon the phrenic nerves to a degree required to establish normal respiration.

"Autopsies have also established that chloroform accumulates in the lungs, but particularly in large quantities in the brain, all parts of which disengage a strong odor of this anæsthetic, which seems to prove that this organ is the place of election of the agent, of which the deadly effects are in proportion to the quantity of the vapor respired."

A discussion followed as to the best method of inflating the lungs in the case in question, it being contended that it was a very difficult thing to do; one member suggesting tracheotomy as the only sure method, and another inquiring how it was possible to introduce the sound into the larynx.

"M. Ferrier replied, that daily experience proves that inflation is less difficult than it is thought to be, and that air blown in by the nasal passages penetrates to the lungs. To introduce the sound, the instrument have been passed to the upper part of the pharynx, it is easy to raise the glottis and slip it into the larynx."

The conclusions of the report were adopted.—*Boston Medical and Surgical Journal.*

